# Atypical modes of COVID-19 transmission: how likely are they?

**DOI:** 10.4178/epih.e2020059

**Published:** 2020-08-11

**Authors:** Viroj Wiwanitkit

**Affiliations:** Dr. D. Y. Patil University, Pune, India

**Keywords:** COVID-19, Transmission, Atypical modes, Public health, Infectious disease

## Abstract

Coronavirus disease 2019 (COVID-19) is a new emerging pandemic, which has so far infected more than 20 million people throughout the world. Typically, this infection is transmitted from humans to humans via respiratory contact. However, the possibility that COVID-19 might be transmitted via atypical modes of transmission is an important public health consideration. In this short review article, the author summarizes and discusses the data on atypical modes of COVID-19 transmission. Based on the available data, it seems that there is still no evidence that COVID-19 can be transmitted via atypical modes of transmission.

## INTRODUCTION

Coronavirus disease 2019 (COVID-19) is a novel coronavirus infection that was first reported in 2019. After its was first detected in China, COVID-19 has become a new emerging pandemic that has caused problems worldwide. The disease has already infected more than 20 million people throughout the world (https://www.worldometers.info/coronavirus/, data at August 11, 2020). Typically, this infection is transmitted from humans to humans via respiratory contact. In this mode of transmission, contaminated respiratory secretions/droplets from an infected person are the vehicle that brings the pathogen to another person [1].

This new respiratory tract infection can result in numerous clinical problems and death. Efforts are being made to control this emerging infectious disease worldwide, but COVID-19 has still not been fully contained. Therefore, the possibility that COVID-19 might be transmitted via atypical modes of transmission is an important public health consideration [2]. In this short review article, the author summarizes and discusses the data on some possible atypical modes of COVID-19 transmission.

## POSSIBILITY OF ATYPICAL MODES OF COVID-19 TRANSMISSION

### Vertical transmission

The possibility of vertical transmission is carefully studied when a new emerging viral infection occurs. Conceptually, if the viral particle is smaller than the barrier of the placenta, the virus might cross the placenta to infect the fetus. Some studies have investigated the possibility of vertical transmission of COVID-19, but no cases of vertical transmission have been reported [3-5]. This is concordant with the fact that severe acute respiratory syndrome coronavirus 2 (SARS-CoV-2), the virus that causes COVID-19, is a large virus, and it therefore is unlikely to cross the placenta to result in vertical transmission. This scenario is similar to that of human immunodeficiency virus vertical transmission [6]. Specifically, transmission should only be possible if pathological changes have taken place in the placenta. The specific issue of possible vertical transmission of COVID-19 is an interesting topic for further research.

### Transmission via breast milk

Infantile COVID-19 has already been recognized. How infants are infected is an interesting question, and in particular, the possibility of COVID-19 transmission via breast milk is an compelling topic in clinical neonatology. As discussed with vertical transmission, if the viral particle is smaller than the breast milk duct, the virus might be secreted via breast milk and might cause infection. However, it appears that SARS-CoV-2 is too large to cross into the breast milk [7]. Therefore, contaminated breast milk from the mother is not expected to cause infections in infants [8], and there is no transmission of COVID-19 via breast milk. Nevertheless, close contact during breastfeeding may be a risk factor for disease transmission. This close contact, without distancing, during breastfeeding might explain why some infants contract COVID-19 from their mothers.

### Blood-borne transmission

Viremia is observable in COVID-19, meaning that the virus can be identified in blood. Although there have not been any confirmed cases, blood banks should implement screening for possible SARS-CoV-2 contamination to ensure the safety of blood and blood products in the present COVID-19 pandemic situation. Nevertheless, viral particles can be detected in convalescent patients for a long time [9]. This is an important issue since interest has emerged in using donated convalescent plasma from patients recovering from COVID-19 to treat COVID-19 patients [10]. It remains unknown whether contaminated convalescent plasma can further cause a new infection [11]. Further studies are required to confirm whether this mode of transmission is feasible.

### Transplantation-related transmission

COVID-19 can occur in transplant patients [12,13], but no report has yet investigated transplantation-related transmission. The virus might be present in internal organs, such as the lungs, and if a contaminated organ is used for transplantation, disease transmission might be possible. Screening for SARS-CoV-2 before transplantation is recommended. Current knowledge on this issue is limited, and it is necessary to collect more information on this topic.

### Vector-borne transmission

COVID-19 can have a similar clinical presentation to many vector-borne diseases, such as dengue [14]. Concurrent epidemics of COVID-19 and other vector-borne diseases are also possible [15]. Nevertheless, there is no evidence that COVID-19 can be transmitted as a vector-borne disease. SARS-CoV-2 has not been observed in mosquitos or other vectors. No transmission occurs through this mode.

### Transmission through sexual contact

COVID-19 is not a sexually transmitted disease, and semen from infected patients has been proven not to be contaminated by SARS-CoV-2 [16]. Therefore, sexual intercourse should be safe, as theoretically, there is no transmission via sexual contact. Nevertheless, sexual intercourse is not a distanced activity and close contact may cause a risk of disease transmission, as was described above regarding transmission via breast milk.

#### Zoonosis

Although it is believed that SARS-CoV-2 is a new virus that crossed to humans from an animal species, the origin of the virus and the identity of patient zero remain unclear. A few reports have documented the existence of the virus in pets such as cats [17,18], but there is still no proof of either human-to-animal or animal-to-human transmission of the new disease. No cases of zoonosis have been confirmed.

### Orofecal transmission

The pathogenic virus might persist in the feces of infected patients for a long time [19]. The chance that environmental contamination from virus-containing feces might cause disease spreading has been widely discussed. A basic caveat regarding this possibility is that the virus is not expected to tolerate gastric acidity, although transmission through contaminated food could be possible. Orofecal transmission of COVID-19 is unlikely, and no instances of orofecal transmission have been confirmed.

### Food-borne and water-borne transmission

These modes of transmission involve similar considerations to the possibility of orofecal transmission of COVID-19. There is no doubt that food or drink might be contaminated by infectious droplets. If there are enough of these droplets, transmission might occur. In fact, there have been some reports of COVID-19 clusters among persons attending a single party and sharing drinks [20]. This might suggest the possibility of disease transmission from contamination by infectious saliva or secretions. It is likely that the virus might be present in the food or drink consumed by an infected person, and might further spread to others who partake of that food or drink. Nevertheless, an important consideration is that the pathogenic virus cannot tolerate gastric acidity, which helps to prevent disease transmission. Interacting with others at a close distance during a party might be a more likely cause of disease transmission in this setting.

### Transmission via skin contact

Skin contact should not be a route through which the pathogen can enter the human body. In particular, contact with an intact skin or wound is not a way that SARS-CoV-2 can be transmitted. However, if the skin comes into contact with a virus-contaminated surface—as a broad range of objects could be contaminated by respiratory secretions—the infection could be transmitted by using one’s contaminated hand to touch one’s nose or mouth, through which the virus can enter the body. Therefore, regular hand washing is recommended to decrease the likelihood of the disease spreading. The virus might also be detectable in several body fluids such as saliva and tears [21]; therefore, routine hand washing is necessary after contact with anyone. Additionally, as a basic hygienic practice, it is recommended not to share any utensils with others. Similarly, if there is a heavily-contaminated aerosol in the environment, infectious particles might drop onto one’s hair or clothing. Therefore, it is recommended to engage in regular showering, hair washing, and thorough cleaning of one’s clothes after being in a crowded area. Although no cases of transmission by this mode have yet been described in the literature, the prevention of contact is still recommended.

### Transmission via smoke exhaled from a smoker

Smoking is considered a health risk behavior. For COVID-19, smoking is also associated with the severity of infection. A report described a cluster of COVID-19 cases who shared cigarettes [22]. Nevertheless, there is still no evidence that there is viral contamination in the smoke exhaled by a smoker. No transmission has been reported by this mode. However, close contact with a smoker is a risk factor for contracting the disease through direct respiratory contact.

### Transmission from a dead body

This possible mode of transmission has also been widely discussed. Many reports have stated that the virus can be isolated from the secretions or organs of dead bodies, and suggested that it might be infectious. However, there still have not been any confirmed cases of transmission of COVID-19 from a dead body [23]. There are many recommendations for safe practices regarding dead bodies and the standard protections that should be applied when coming into contact with a corpse [23]. A single report has described a case of COVID-19 infection in a forensic staff member who worked closely with dead bodies in his daily routine, but it was not possible to conclude whether that patient contracted the disease from occupational exposure to dead bodies or other non-occupational contact in daily life activities [23]. We do not currently know whether COVID-19 can be transmitted from dead bodies, but prevention is necessary.

## CONCLUSION

Atypical modes of COVID-19 transmission are an interesting issue in global public health. Based on the available data, it seems that there is still no evidence that COVID-19 can be transmitted via atypical modes of transmission (Table 1). From a logical standpoint, contaminated objects might be a possible source for spreading of the virus. Contact with any contaminated object might cause disease transmission, although contact with contaminated food and drink should be more intense for transmission to be likely. The other important modes of transmission, including zoonosis, sexual transmission, breast milk transmission, and vertical transmission, should not be possible. Since COVID-19 is a new disease, it is necessary for future studies to verify the possibility of atypical modes of transmission.

### Ethics statement

This paper is a perspective so it did not need ethical consideration.

## Figures and Tables

**Table 1.** Summary of the transmission modes of coronavirus disease 2019 (COVID-19) in comparison to other important infectious diseases

Mode	HBV	TB	HIV	Influenza	COVID-19 transmission
Human to human by respiratory contact: droplets	No	Yes	No	Yes	It has been confirmed that contaminated droplets are the main source of virus spread
Human to human by respiratory contact: airborne	No	Yes	No	No	It remains controversial whether COVID-19 is an airborne infection and this possibility requires further study
Vertical transmission	Yes	Yes	Yes	No	No report, and it should not be possible
Transmission via breast milk	No	No	Yes	No	No report, and it should not be possible
Blood-borne transmission	Yes	No	Yes	No	No report, but further studies are required
Transplantation-related transmission	Yes	No	Yes	No	No report, but further studies are required
Vector-borne	No	No	No	No	No report, and it should not be possible
Sexual contact	Yes	No	Yes	No	No report, and it should not be possible
Zoonosis	No	No	No	No	No report, but further studies are required
Orofecal transmission	No	No	No	No	No report, but further studies are required
Food-borne	No	No	No	No	No report, but contact with contaminated food might be a possible cause of transmission
Water-borne	No	No	No	No	No report, but contact with contaminated drink might be a possible cause of transmission
Skin contact	No	No	No	No	No report, and it should not be possible
Exhalation from smoker	No	Possible^1^	No	No	No report, but further studies are required
Contact with dead bodies	Possible^1^	Possible^1^	Possible^1^	No	No report, but it might be possible

HBV, hepatitis B virus infection; TB, tuberculosis; HIV, human immunodeficiency virus infection.

1 Some possible incidents without conclusive scientific confirmation.
